# Equilibrium, Thermodynamics, and Kinetic Sorption Studies for the Removal of Coomassie Brilliant Blue on Wheat Bran as a Low-Cost Adsorbent

**DOI:** 10.1155/2012/405980

**Published:** 2012-03-27

**Authors:** Sadia Ata, Muhammad Imran Din, Atta Rasool, Imran Qasim, Ijaz Ul Mohsin

**Affiliations:** ^1^Institute of Chemistry, University of Punjab, Lahore, Pakistan; ^2^Institute of Chemical Technology & Analytics, Vienna University of Technology, 1060 Wien, Austria

## Abstract

The sorption studies of coomassie brilliant blue (CBB) from aqueous solution have been carried out on wheat bran (WB). Coomassie brilliant blue on wheat bran was used to study the adsorption behavior under various parameters such as pH, dosage amount, and contact time. It was observed that under optimized conditions up to 95.70% dye could be removed from solution onto WB. Langmuir and Freundlich adsorption isotherms were used to elaborate the results. Freundlich model was found to be fitted well and favored multilayer adsorption. The Freundlich constants n and KF were determined as 0.53 and 2.5 × 10^−4^. Thermodynamic parameters such as Δ*G*, Δ*H*, and Δ*S* studied were taking into account, showed spontaneous and favorable reaction for coomassie brilliant blue on wheat bran. The maximum adsorption capacity *q*_*m*_ was found to be 6.410 mg/g. The investigations show that non treated WB is a low-cost adsorbent for the removal of dyes from textile industry effluents.

## 1. Introduction

The inappropriate disposal of dyes in waste water constitutes an environmental problem and can cause damage to the ecosystem [1]. Water pollution by dyes is one of the major pollution sources. Wastewater containing dyes cause water pollution by lowering light penetration and photosynthesis and toxicity from heavy metals associated with pigments [2]. Synthetic dyes are widely used in industries such as textiles, leather, paper, and plastics to color final products. Reactive dyes are the most common dyes used due to their advantages, such as bright colors, excellent colorfastness, and ease of application but effluents are highly toxic to aquatic life. It reduces the photosynthetic activity and primary production. These dyes are mostly resistant to biodegradation and therefore are not removed by conventional treatment techniques [3]. Dyes are colored substances which colored a substrate as they retained in it by adsorption. These are soluble completely or partially soluble in a media from which they are applied in contrast with pigments. Dyes must possess a specific affinity to the substrate from which they are used. Some of the dyes are either toxic or carcinogenic. Synthetic dyes have complex aromatic structures which provide them physiochemical, thermal, biological, and optical stability [4] and used in photographic paper as indicator and as biological stains. Commercial uses of dyes include the coloration of textile paper, leather, wood, inks, fuels, food items and metals.

This dye may cause problems in, respiratory tract, gastrointestinal tract, irritation to skin, and redness of eyes. It may cause adverse effects on ecoquatic system [5, 6].

Prasad et al. studied the biosorption of coomassie brilliant blue by acid treated coir pith in batch mode. Effect of time, initial concentration of dye and initial adsorbent concentration on biosorption was investigated. The maximum adsorption capacity was found to be 31.847 mg/g. The investigation shows that the acid-treated coir pith is better suited for the removal of dyes from the textile industry effluent [7]. 

Katarzyna and Watala studied a new photometric method for determining the surface area of plants, which relies on the adsorption of coomassie brilliant blue G-250 on the plant surfaces. The method was applied to a variety of aquatic plants and found to provide a routine procedure to determine plant surface area [8].

Norie et al. studied Okara quickly adsorbed coomassie brilliant blue (CBB) stain. Compared to activated carbon and/or carbon fiber adsorption, in this research, the characteristics and mechanism of adsorption were examined with the aim to develop an efficient dyestuff adsorption system using Okara. The amount of the saturation dye adsorption, *Q*_*m*_ (mg-dye/g-okara) obtained from the Langmuir adsorption isotherm after 24 h at pH 3 was 197. It was found that Okara's adsorption ability is approximately 66 times higher than the ability of activated carbon. The adsorption efficiency of the dye is influenced by pH [9].

Rauf et al. studied three organic dyes namely, coomassie blue, Malachite Green and Safranin Orange by adsorption on sand at 298 K. Characteristics of local sand sample used as an adsorbent in this work were initially found from the low-temperature adsorption of nitrogen on sand samples at 77 K. Conditions for maximum adsorption of these dyes on sand sample were then optimized. The adsorption behavior of the dyes was also investigated in terms of added cations and anions and it was found that adsorption of coomassie blue and safranin orange decreased substantially in the presence of sulphate, thiosulphate, acetate, potassium, and nickel and zinc ions [10].

Wei et al. studied the interactions of coomassie brilliant blue G-250 (CBB) with bovine serum albumin (BSA) and 7-globulin at low pH that are investigated by a spectrophotometric method. It is considered that the binding of CBB to protein is because of the weak interactions (ionic, van dar Waals, hydrogen bonding, and hydrophobic). Factors which influence the sensitivity of the CBB protein assay are studied using this method. Ionic strength and acidity are found to have significant effect on the binding of CBB to protein [11].

The objective of the present study was to assess the ability of locally available Bran for the removal of coomassie brilliant blue (CBB). The study was carried out with the aim to optimize conditions for maximum removal of this dye from aqueous solutions by using wheat bran. Besides this, the adsorption data was fitted to various equations to obtain constants related to the equilibrium of the adsorption phenomena. The removal of CBB was about 98% using wheat bran.

## 2. Materials and Experimental Procedure

### 2.1. Reagents and Solutions

Pure coomassie brilliant blue R-250 was used for the preparation of standard coomassie brilliant blue R-250 stock solution. Distilled water was used as a solvent. The main characteristics of Comassie Brilliant Blue are and *λ*_max⁡_ = 580 nm, Color Index (C.I) = 42660, Chemical formula = C_45_H_44_N_3_NaO_7_S_2_, and M.W. = 825.97 g/mol and is insoluble in cold water and slightly soluble in hot water.

### 2.2. Preparation of Adsorbent

Wheat has a hard outer layer. When it is processed, this layer becomes a byproduct, and is called bran. In the case of processing wheat to make wheat flour, one gets millers or wheat bran. It is collected and dried in direct sunlight for three days. After three days wheat bran was put in oven at 60°C for twenty four hours. Dried wheat bran was thoroughly washed with distilled water to remove all dirt and was dried at 100°C till constant weight. Then the dried wheat bran was grinded and sieved with a 30-mesh sieve, to prepare homogenous particle size. This biosorbent was again washed with distilled water to remove adhearing dirt and foreign particles. Then, it was kept in oven at 60°C for 24 hours. The average particle size of adsorbents was measured and it was 500 *μ*m and BET surface area was measured by the Brunauer, Emmett and Teller (BET) method using N_2_ gas adsorption (Quanta-chrome Inst., Nova 2200e Surface Area Analyzer) and it was calculated as 8.62 m^2^/g. 

### 2.3. Comassie Brilliant Blue Stock Solution

100 mg/L Comassie Brilliant Blue stock solution was prepared in distilled water and stored in brown reagent bottles in dark place to prevent oxidation of the dye solution.

Various concentrations of Comassie Brilliant Blue solutions ranging from 1 to 10 ppm were prepared from the Comassie Brilliant Blue stock solution in 100 mL measuring flasks. Then volume was up to the mark with distilled water. At the start the color of the solution was violet blue.

**Figure 1:**
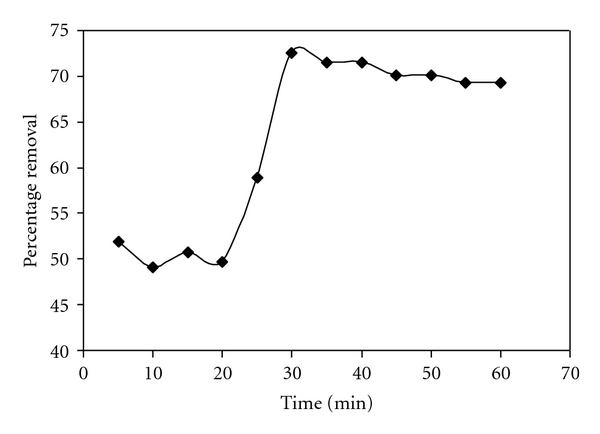
Effect of contact time on adsorption of CBB by wheat bran.

With the help of double beam spectrophotometer absorbance of Comassie Brilliant Blue was measured at wavelength of 580 nm in a 1.0 cm matched quartz curettes against a reagent blank (without soln.) on through mixing. At this wavelength all the experiments were performed. Beer Lambert law was obeyed. This general method was adopted for the rest of the experiments. The values obtained from this experiment were given in Table 1 and graph shown in Figure 1.

The adsorption of Comassie Brilliant Blue on wheat bran was studied by a batch technique. The general method used for these studies is described below.

0.1 g of the dried sieved wheat bran was equilibrated with 10 cm^3^ of the Comassie Brilliant Blue solution of the known concentration in a stopper Pyrex glass flasks at fixed temperature in a shaker for a known period of time. The flasks containing the weighed amounts of wheat bran and Comassie Brilliant Blue solution were separately kept in the before mixing for a sufficient period of time to attain the desired experimental temperature. After equilibration the suspension was centrifuged in a stopper tube for 10 minutes at 3500 rpm. The concentration of Comassie Brilliant Blue in a known volume of the supernatant was determined by spectrophotometrically. The amount of Comassie Brilliant Blue adsorbed on the wheat bran was thus determined. Adsorption of Comassie Brilliant Blue on wheat bran was determined in terms of equilibrium concentration (*C*_*e*_), percentage Removal *P*%, Adsorption Capacity *q*_*e*_ determined as, the equilibrium concentration (*C*_*e*_) is determined.

The percentage adsorption *P* was calculated. The amount adsorbed per unit weight of the wheat bran, *x*/*m*, was calculated spectrophotometrically from the initial (known Comassie Brilliant Blue concentrations) and final absorbance of the solutions.

### 2.4. Effect of Contact Time

The adsorption of C.B.B on wheat was studied as a function of shaking time at 35°C. A sample of 50 cm^3^ of C.B.B (10 ppm) solution was taken in nine titration flasks, labeled them from 1–9, and shaken with 0.1 g of wheat bran in each flask for different intervals of time ranging from 5 min to 60 min in a shaker. After equilibration the suspension was centrifuged for at 3500 rpm.

After 15 minutes all the solutions were filtered and absorbance was determined photometrically at wavelength 560 nm. From it, calculate the equilibrium concentration (*C*_*e*_) of C.B.B, percentage removal, and equilibrium time was determined by plotting a graph between different time intervals and percentage removal_._

Figure 1 shows the variation of % age adsorption as a function of shaking time. The result shows that the adsorption of C.B.B on wheat bran is symmetrically time dependant which increases with the shaking time rapidly and gets to an equilibrium stage after 30 minutes which dose not change latter on. So for all other experiments shaking time was kept 30 minutes. The values of equilibrium concentration (*C*_*e*_) and % age adsorption were shown in Figure 1.

As adsorption and desorption occur side by side so after an appropriate time equilibrium takes place. Composition of wheat bran also plays a major role in this regard. At 30 minute, the maximum adsorption is observed due to the attractive forces developed between CBB and WB.

Initial adsorption of dye is on exterior surface of wheat bran. After complete adsorption on outer surface, the dye enters to the inner surface via pores. After 30 minutes desorption takes place.

### 2.5. Effect of pH

The adsorption of C.B.B on wheat bran was studied as a function of different pH. 1–10, while keeping all other parameters, that are, C.B.B concentration (10 ppm), shaking time (30 min). After equilibration, the suspension was centrifuged for 30 minutes at 3500 rpm. After 15 minutes all the solutions were filtered and absorbance was determined photometrically at wavelength 560 nm.

**Figure 2:**
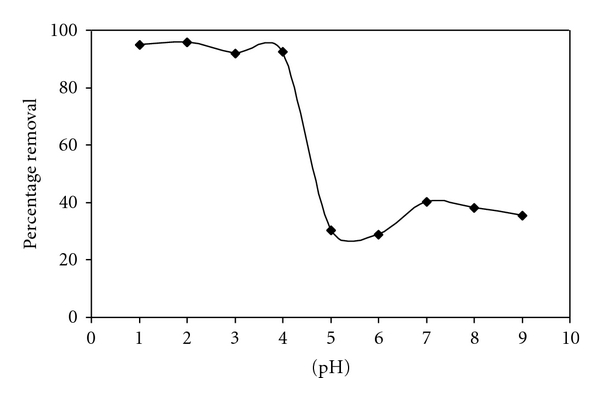
Effect of pH on adsorption of CBB by wheat bran.

From it, calculate the amount of equilibrium concentration (*C*_*e*_) of C.B.B, percentage removal, and determined the adsorption of C.B.B on wheat bran at different pH by plotting a graph between different pH and percentage removal.  Figure 2 shows the variation of equilibrium concentration (*C*_*e*_) and percentage removal as a function of different pH. The result shows that the adsorption of C.B.B on wheat bran increases with increasing pH and attains to maximum at pH 2.0. Both *C*_*e*_ and % age adsorption also increase with increasing pH till pH 2.0 and then decreased sharply. Therefore, other experiments were carried out at pH 2.0. The values of equilibrium concentration (*C*_*e*_) and % age adsorption were shown in Figure 2.

Adsorption is greater at lower pH. It may be due to the increased positive charges on the surface of adsorbent that attracts the functional groups of a dye carrying negative charge.

### 2.6. Effect of Adsorbent Dosage

The adsorption of C.B.B on wheat bran was studied as a function of different amount of adsorbent, that is, 0.1–1.0 g, while keeping all other parameters that is, C.B.B concentration (10 ppm), shaking time (30 min), and pH (2.0), of the suspension constant.

**Figure 3:**
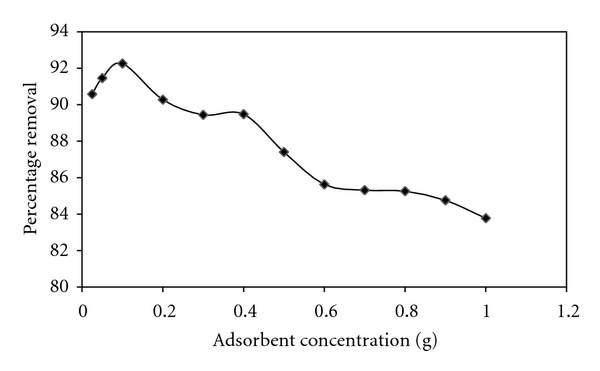
Effect of amount of adsorbent on adsorption of CBB by wheat bran.

Calculate the equilibrium concentration (*C*_*e*_) of C.B.B and % age adsorption and determine the adsorption of C.B.B on wheat bran at different amount of adsorbent by plotting a graph between different amount of adsorbent and % age adsorption.  Figure 3 shows the variation of % age adsorption as a function of different amount of adsorbent. The results show that the adsorption of C.B.B on wheat bran increases spontaneously with increasing adsorbent concentration due to availability of greater active sites. % age adsorption also increased linearly with increasing adsorbent concentration. On the basis of these results, 0.1 g/50 cm^3^ of C.B.B solution was selected for further studies. The values of equilibrium concentration (*C*_*e*_) and % age adsorption were shown in Figure 3. The decrease in % age removal of dye with increased amount of adsorbent is due to the fact that adsorption sites remain unsaturated during adsorption reaction.

### 2.7. Effect of Sorbate Concentration

**Figure 4:**
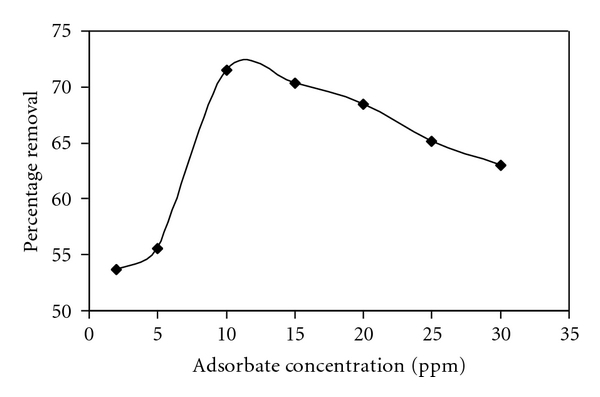
Effect of concentration of dye on adsorption of CBB by wheat bran.

The adsorption of C.B.B on wheat bran was studied as a function of its different concentrations, that is, 5–30 ppm, while keeping all other parameters that is, shaking time (30 min) and pH (2.0), sorbent concentration (0.1 g) constant. Calculate the equilibrium concentration (*C*_*e*_) of C.B.B and % age adsorption and determined the adsorption of C.B.B on wheat bran at its different concentrations by plotting a graph between different concentration of sorbate and % age adsorption.  Figure 4 shows the variation of % age adsorption as a function of different sorbate concentrations. The results show that the adsorption of C.B.B on wheat bran increases spontaneously with increasing sorbate concentration till 30 ppm and then become constant onward. % age adsorption increases continuously with increasing C.B.B concentration. On the basis of these results 0.2 g/1000 cm^3^ of C.B.B solution was selected for further studies. The values of equilibrium concentration (*C*_*e*_) and % age adsorption were shown in Figure 4.

### 2.8. Effect of Temperature

**Figure 5:**
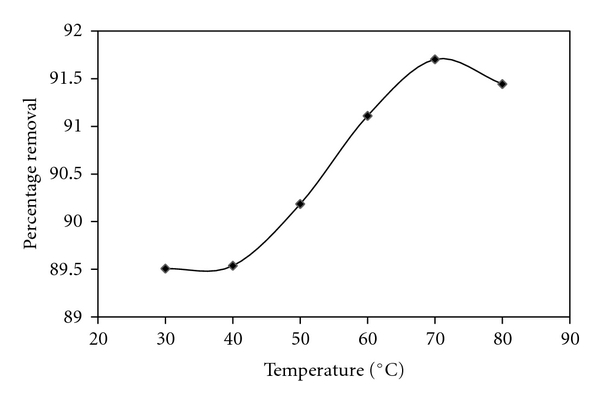
Effect of temperature on adsorption of CBB by wheat bran.

The adsorption of C.B.B on wheat bran was studied as a function of different temperature, that are, 30°C, 40°C, 50°C, 60°C, 70°C, and 80°C, while keeping all other parameters that are, C.B.B concentration (10 ppm), shaking time (30 min), pH (2.0), and sorbent concentration (0.1 g), constant. From it calculate the equilibrium concentration (*C*_*e*_) of C.B.B and % age adsorption and determined the effect of temperature on adsorption of C.B.B on wheat bran by plotting a graph between different temperature and % age adsorption.  Figure 5 shows the variation of % age adsorption as a function of different temperature. The results show that the adsorption of C.B.B on wheat bran was increased spontaneously with increasing temperature. % age adsorption also increased linearly with increasing temperature till 60°C then decreases. The values of equilibrium concentration (*C*_*e*_) and % age adsorption were shown in Figure 5. Increase in adsorption at elevated temperature is due to the enlargement of pore size from where dye adsorbed.

### 2.9. Adsorption Isotherm

The adsorption of C.B.B on wheat bran was studied as a function of different C.B.B concentration that is, 5–30 ppm, while keeping all other parameters that are, shaking time (30 min), pH (2.0), and sorbent concentration (0.1 g) constant. 50 cm^3^ of C.B.B (5 ppm to 30 ppm) solution was shaken with 0.1 g of wheat bran for 30 min in a shaker. After 15 minutes, the absorbance was determined photometrically at wavelength 560 nm. Then calculated the adsorbed amount of C.B.B and equilibrium concentration (*C*_*e*_) and determine the adsorption isotherms C.B.B on wheat bran by plotting a graph between extent of adsorption (log⁡*q*) and equilibrium concentration log *C*_*e*_ (moldm^−3^) [12]. 

There are two types of adsorption isotherm:

- *Langmuir Adsorption Isotherm*.
- *Freundlich Adsorption Isotherm*.

The adsorption isotherms were used to investigate the relationship between the concentration of sorbed species and sorption capacity of sorbing species.

**Langmuir Adsorption Isotherm**

Linear form of Langmuir adsorption isotherms is shown as follows [13]:

(1) $$\frac{1}{q_{e}}=\frac{1}{Q_{\max}\;}+\frac{1}{b\cdot Q_{\max}}\cdot \frac{1}{C_{e}}$$

  *q*_*e*_: sorption capacity, *C*_*e*_: equilibrium concentration, *Q*_max⁡_: maximum possible amount of dye that can be adsorbed per unit dry weight of sorbent, *b*: Empirical constant, indicating the affinity of sorbent towards the sorbate.

**Figure 6:**
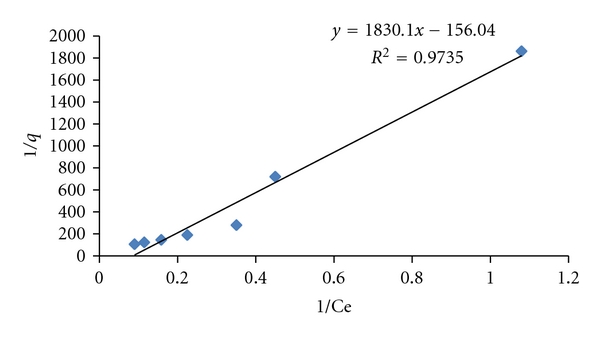
Graph between 1/*C*_*e*_ and 1/*q*_*e*_ for Langmuir Adsorption Isotherm.

A curve 1/*q*_*e*_ versus 1/*C*_*e*_ has been plotted to investigate the fitting of Langmuir model for the equilibrium data of WB-dye sorption a straight line was obtained. The correlation coefficient *R* ^2^ was found to be 0.9732 indicating that the data was fitted well the Langmuir model (Figure 6). 

From the Langmuir plot, *Q*_max⁡_ = 6.410 mg/g and *b* = 3.502 L/mg. The characteristics of the Langmuir isotherm can be expressed as another constant separation factor or equilibrium parameter given by *R*_*L*_ [14].

(2) $$R_{L}=\frac{1}{1+K_{L}\cdot C_{\text{o}}},$$

where *K*_*L*_ is Langmuir's Equilibrium Constant which is related to the affinity of binding sites and *K*_*L*_ = *Q*_max⁡_ · *b*. *C*_o_ is the initial dye concentration. According to McKay et al. *R*_*L*_ between 0 and 1 indicates favorable adsorption. In the current experiment *R*_*L*_ is found to be 0.0042636 and again the adsorption is found to be favorable [15].

**Freundlich Absorption Isotherm**

**Figure 7:**
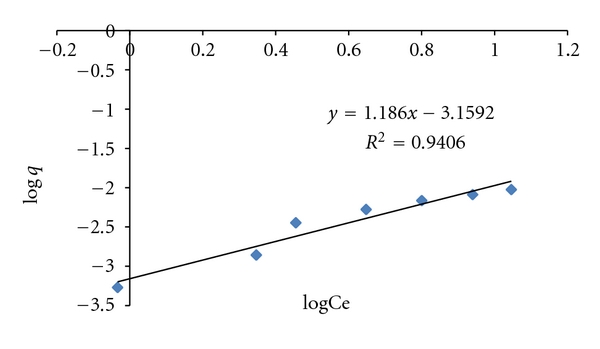
Graph between log⁡*C*_*e*_ and log⁡*q*_*e*_ for Freundlich absorption isotherm.

Linear form of Freundlich adsorption isotherms is shown as [16–18].

(3) $$\log q_{e}=\log K_{f}+\frac{1}{n}\log C_{e},$$

where *q*_*e*_ is the equilibrium dye concentration sorbed in sorbent (mg g^−1^), “*C*_*e*_” is the dye equilibrium concentration in solution (mg L^−1^). *K*_*f*_ is Freundlich constant related to the sorption capacity, and 1/*n* is an empirical parameter related to sorption intensity, which varies with heterogeneity of the material. The equilibrium data was also used to investigate Freundlich model. A plot of log *q*_*e*_ versus log *C*_*e*_ was obtained to study the model. The correlation factor *R* ^2^ was 0.9400 that data also fit the Freundlich model. The values of *k*_*f*_ and *n* were 2.5 × 10^−4^ and 0.53. The value of *n* is between 0–1 indicating favorable sorption of CBB [19] (Figure 7).

#### 2.9.1. Dubnin-R Equation

This equation is used to determine sorption mean energy which enabled us to estimate whether adsorption is carried out by ion exchange mechanism, physisorption or, chemisorptions [20].

(4) $$\begin{array}{c} \ln q_{e}=\ln q_{m}-\beta \varepsilon^{2},\\ \varepsilon =RT\ln\left(1+\frac{1}{C_{e}}\right), \end{array}$$

where *q*_*m*_ is a theoretical saturation capacity, *β* is a constant related with sorption energy, mol^2^J^−2^, *ε* is a polonyi potential and is equal to *RT*ln⁡(1 + 1/*C*_*e*_). Mean sorption energy *E* is determined by

(5) $$E_{s}=\frac{1}{\sqrt{2\beta }}.$$

**Figure 8:**
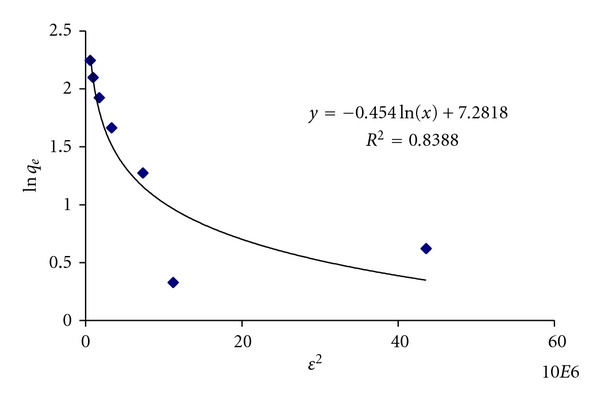
Graph between polonyi potential *ε* and ln⁡*q*_*e*_ for activation energy.

*β* is calculated from slope of a linear graph ln⁡*q*_*e*_ versus *ε* ^2^ shown in Figure 8. 

The value of sorption mean energy is 0.26 KJmol^−1^. It is positive and less than 8 KJmol^−1^. This shows that there is a physisorption and ion exchange. 

### 2.10. Thermodynamic Parameters

The effect of a change in temperature on the sorption system was studied to determine the thermodynamic parameter and to investigate the nature of the process. The sorption capacity increases with the temperature. Thermodynamic parameters for adsorption of C.B.B on wheat bran such as heat of adsorption (enthalpy change, Δ*H*), entropy change, Δ*S*, free energy of specific adsorption and Δ*G* were calculated from the binding constant *K*_*c*_ obtained from Langmuir plot by using the following equation.

(6) $$\Delta G^{\text{o}}=\Delta H^{\text{o}}-T\Delta S^{\text{o}}.$$

below is the equation of straight line:

(7) $$\begin{array}{c} \Delta G^{\text{o}}=-RT\ln K_{D},\\ K_{D}=\frac{q_{e}}{C_{e}},\\ \ln K_{D}=-\frac{\Delta H^{\text{o}}}{2.303RT}+\frac{\Delta S^{\text{o}}}{2.303R}. \end{array}$$

For activation energy, following equation is used

(8) $$\ln K=\ln A-\frac{E_{a}}{2.303RT}.$$

*E*_*a*_ can be calculated from slope of a graph ln⁡*K* versus 1/*T* as shown in Figure 10. 

**Figure 9:**
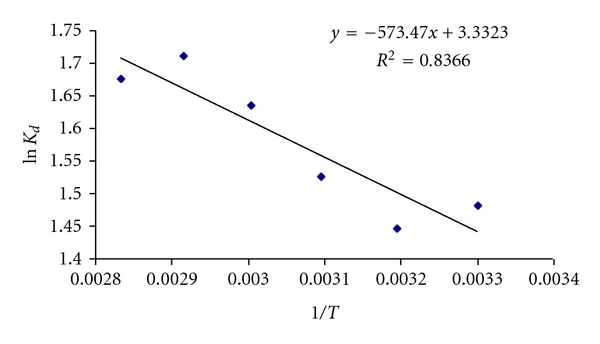
Graph between 1/*T* and ln⁡*k*_*d*_.

**Figure 10:**
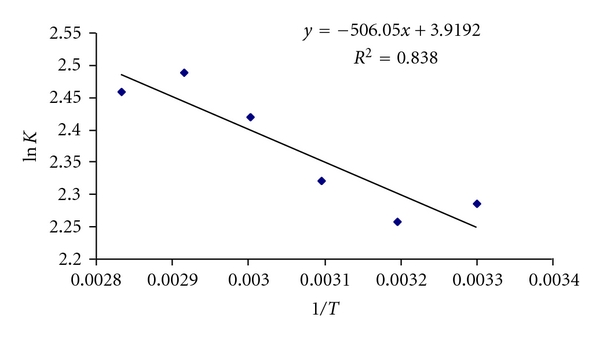
Graph between 1/*T* and ln⁡*K*.

Where *R* is the gas constant, *K*_*c*_ is the equilibrium constant, *T* is the temperature (*K*), *C*_o_ is the equilibrium concentration of C.B.B on adsorbent (mg/L), and *C*_*e*_ is the equilibrium concentration of C.B.B in the solution (mg/L). The values of Δ*H* ^o^ and Δ*S* ^o^ were calculated from the slope and intercept. The values of thermodynamic parameters Δ*H* and Δ*S* for the C.B.B. on wheat bran calculated from above equations were shown in Figure 9 and listed in Table 2. 

The negative values of Δ*G* indicates the spontaneous nature of adsorption. As the temperature increases, the Δ*G* values increase, indicating more driving force and hence resulting greater adsorption capacity at higher temperatures. The positive values of Δ*H* and *E*_*a*_ confirm the endothermic nature. 

### 2.11. Kinetic Parameters

The Lagergren^,^s kinetics equation has been most widely used for the adsorption of an adsorbate from an aqueous solution. The Lagergren model may not reflect the true nature of kinetic study. It was applied due to its simplicity and good fit [21]. 

The model is based on the assumption that rate is directly proportional to the number of free sites according to Lagergren equation:

(9) $$\log\left(q_{e}-q_{t}\right)=\log q_{e}-\frac{K_{t}}{2.303}.$$

**Figure 11:**
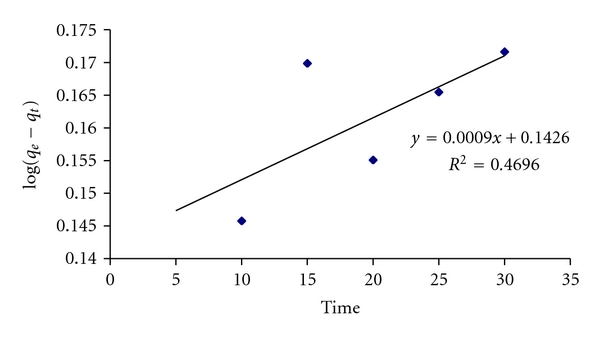
First-order adsorption kinetics of CBB on WB.

Linear plot of log (*q*_*e*_ − *q*_*t*_) versus *t* was plotted to evaluate this kinetic model and to determine rate constant *K*. The values of *R* ^2^ and *K* are 0.469 and 0.002073, respectively, shown in Figure 11. It depicts that Lagergren model is not fitted in sorption of CBB on WB. 

The pseudo-second-order model is based on the biosorption followed by second-order mechanism nearby the rate of sorption is proportional to the square of number of unoccupied sites and can be represented as

(10) $$\frac{t}{q}=\frac{1}{K_{2}q_{e}^{2}}+\frac{t}{q_{t}}.$$

**Figure 12:**
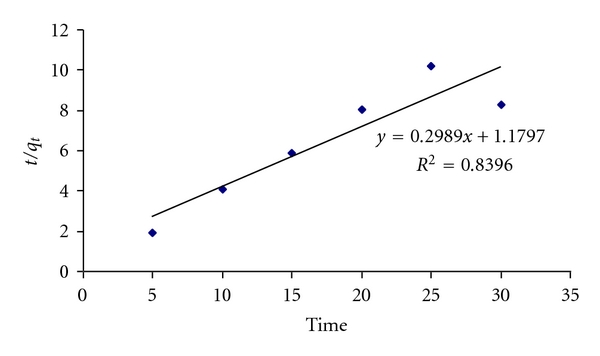
Pseudo-second-order adsorption kinetics of CBB on WB.

The parameters *q*_*e*_ and *K*_2_ are calculated from slope and intercept of a plot *t*/*q*_*e*_ versus time shown in Figure 12. The value of *R* ^2^ is 0.8396 that shows pseudo-second-order model is best fitted.

## 3. Conclusion

The result of this study indicates that WB is an effective sorbent for the uptake of toxic dye from aqueous solution. The optimum parameters for equilibrium study are time of contact 30 min, pH 2, and dose of W.B 2 gL^−1^ under optimum conditions. The sorption of CBB dye by WB followed a monolayer sorption model Langmuir isotherm rather than multilayer model. Thermodynamic parameters like Δ*G* ^o^, Δ*H* ^o^, and Δ*S* ^o^ were also determined. Their values indicate the spontaneous nature of sorption. Enthalpy values showed that sorption of dye is an endothermic process. WB is easily available, low-cost, and biodegradable. Therefore, WB has been proved to be cost effective sorbent for CBB sorption.

## Figures and Tables

**Table 1:** Langmuir constants for C.B.B adsorption on wheat bran.

Slope	Intercept	*R* ^2^	*Q*_max⁡_	*b*
1/*b*, *Q*_*m*_	1/*Q*_*m*_		1/intercept	1/slope, *Q*_*m*_
1.830	−0.156	0.973	6.410	3.502

**Table 2:** Thermodynamic parameters for the C.B.B. on wheat bran at different temperatures.

Temperature *T* (K)	Thermodynamic parameters			
	Δ*G* (KJmol^−1^)	Δ*S* (kJ/mol k)	Δ*H* (KJmol^−1^)	Activation energy *E*_*a*_
303	−5756.13			
313	−5871.38			
323	−6230.61	1320.7	63.77	9684.75
333	−6697.71			
343	−7095.5			
353	−7212.1			
